# Effect of Sn Addition on Microstructure, Aging Properties and Softening Resistance of Cu-Cr Alloy

**DOI:** 10.3390/ma15238441

**Published:** 2022-11-27

**Authors:** Gaolei Xu, Yunqing Zhu, Lijun Peng, Haofeng Xie, Zengde Li, Shuhui Huang, Zhen Yang, Wenjing Zhang, Xujun Mi

**Affiliations:** 1 State Key Laboratory of Nonferrous Metals and Processes, GRIMAT Group Co., Ltd., Beijing 100088, China; 2 GRIMAT Engineering Institute Co., Ltd., Beijing 101407, China; 3 Zhejiang Libo Industrial Co., Ltd., Shaoxing 312050, China; 4 College of Advanced Copper Industry, Jiangxi University of Science and Technology, Yingtan 335000, China; 5 Jiangxi Libo Kecheng Copper Industry Co., Ltd., Yingtan 335000, China; 6 General Research Institute for Nonferrous Metals, Beijing 100088, China

**Keywords:** Cu-Cr-Sn alloy, microstructure evolution, aging treatment, softening resistance

## Abstract

The relationship between microstructure evolution and properties of a Cu-Cr-Sn alloy during aging and high-temperature softening was investigated in detail in the present work. The results show that the addition of Sn refines obviously the size of the Cr phase and enhances the thermal stability of the alloy, which improves the peak-aged hardness of the Cu-Cr-Sn alloy reaching 139 HV after aging at 450 °C for 240 min. In addition, the recrystallization behavior of the Cu-Cr alloy with the 0.12 wt.% of Sn at high temperature is also significantly inhibited. Lots of precipitated Cr phases and a high density of dislocations are found in the Cu-Cr-Sn alloy annealed at high temperature, resulting in the softening temperature of the Cu-Cr-Sn alloy reaching 565 °C, which is higher than (about 50 °C) that of the Cu-Cr alloy.

## 1. Introduction

Cu-Cr alloy possesses high strength and high electrical conductivity, which has been widely used in high-speed rail contact wires, lead frames, heat exchange, and so on [1,2]. Micro-alloying [3] and thermal-mechanical treatment [4,5] are extremely significant routes to further improve the comprehensive properties of the Cu-Cr alloy. The addition of the Zr element can effectively promote the precipitation of the Cr phase and the fine rich-Zr phases (such as Cu_4_Zr and Cu_5_Zr) to enhance the strength and softening resistance [6,7]. Although the excellent properties of Cu-Cr-Zr have been explored for a long time, it is still difficult to effectively control the uniform composition of the zirconium element because Zr is easily oxidized during the melting process. Therefore, researchers are constantly exploring new alternatives to the Zr element [8,9].

Among them, some non-Zr elements, such as Mg [10], Ag [11], Ti [12,13], and Y [14,15], can also be used to control the microstructure and improve the properties of the Cu-Cr alloy. Mg addition can inhibit the coarsening of the precipitated phase by enrichment at the interface of Cr precipitates through Mg atoms, which can enhance the mechanical properties and softening resistance of the alloy [16,17]. Ag dissolves in the Cu matrix and can increase the strength of the Cu-Cr alloy by solution strengthening without reducing the electrical conductivity [8,18]. The addition of Ti can promote the precipitation of the Cr phase, inhibiting the transition from fcc Cr-rich precipitates to bcc structure [9,13].

In addition, lower-cost Sn microalloying can greatly improve the strength of the Cu-Cr alloy [19]. Li et al. [20] found the tensile strength and electrical conductivity of the 80% cold-rolling Cu-0.67Cr-0.23Sn alloy were increased to 566.3 MPa and 73.3% IACS during aging at 400 °C for 120 min. Luo et al. [21] obtained that the activation energy of recrystallization of the 60% cold-drawn Cu-0.48Cr-0.16Sn alloy is about 117.9 kJ/mol. In this work, the influence mechanism of Sn addition on microstructure and property evolution was further studied. The softening temperature of the Cu-Cr-Sn alloy was measured, and the softening behavior and mechanism of the Cu-Cr-Sn alloy were clarified as well.

## 2. Materials and Methods

Raw materials in the present work were high-purity copper, Cu-20 wt.%Cr master alloy, and pure tin. Cu-Cr and Cu-Cr-Sn alloys were melted at 1250 °C in a vacuum medium-frequency induction furnace (Shanghai Chenhua Technology Co., Ltd., Shanghai, China). The compositions of both alloys (as shown in Table 1) were measured by inductively coupled plasma emission spectroscopy. After being cleaned, both alloys were subjected to solution treatment at 900 °C for 12 h followed by water quenching and cold drawn by 99% deformation. The cold-deformed alloys were aging treated at 400 °C and 450 °C with different holding times (0–960 min), respectively. Both peak-aged alloys were annealed at 300~700 °C for 60 min, and the softening temperature of the two alloys was measured according to national standard GB/T 33370-2016.

The microhardness was measured with a 430 SVD-type Vickers hardness tester (Beijing Huahai Henghui Technology Co., ltd., Beijing, China) using a 5 kg load for 20 s (10 measurements were performed per sample). The electrical conductivity was measured with a TH2512B Intelligent DC low resistance tester (3 measurements were performed per sample) (ChangZhou TonghuiTechnology Co., Ltd., Changzhou, China). The microstructure evolution of both alloys with different annealing treatments was observed by optical microscopy (OM) (Guangdong Jingpu Industrial Co., Ltd., Guangdong, China) and with a transmission electron microscope (TEM) (FEI, Hillsboro, OR, USA)). The samples for TEM observations were mechanically thinned into 50 µm and stamped to 3 mm diameter circle sheets, obtained with a twin-jet unit with 25% HNO_3_ and CH_3_OH below about −40 °C.

## 3. Results

### 3.1. Properties

**Figure 1:**
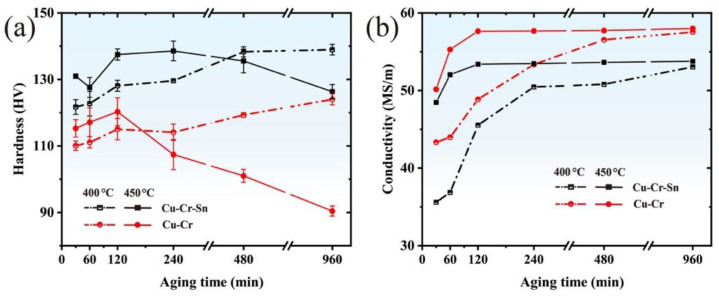
(**a**) Hardness and (**b**) electrical conductivity of Cu-Cr and Cu-Cr-Sn alloys aged at different temperatures.

Figure 1a shows the microhardness curves of the Cu-Cr and Cu-Cr-Sn alloys during aging at different temperatures. The hardness of the Cu-Cr-Sn alloy is much higher than the Cu-Cr alloy at 400 °C and 450 °C by Sn addition, respectively. During aging treatment at 400 °C, the hardness of both alloys increased continuously with the extension of aging time, and the Cu-Cr-Sn alloy reached 138 HV after annealing for 960 min. During aging at 450 °C, the peak hardness of the Cu-Cr and Cu-Cr-Sn alloys reached 120 HV and 139 HV after holding for 120 min and 240 min, respectively. While the hardness of both alloys decreased to a certain extent during the over-aging stage at 450 °C, the addition of Sn can also obviously enhance the thermal stability of the alloy. After aging at 450 °C for 960 min, the hardness of the Cu-Cr-Sn alloy decreases by less than 10% from the peak-aged hardness and is about 36 HV higher than the Cu-Cr alloy.

The electrical conductivity curves of the Cu-Cr and Cu-Cr-Sn alloys are shown in Figure 1b during 400 °C and 450 °C aging treatment. The conductivity of the two alloys increases continuously with the extension of aging time and tends to be constant at the over-aging stage. When aged for 960 min, the electrical conductivity of the Cu-Cr and Cu-Cr-Sn alloys with the two aging temperatures are stable at 57 MS/m and 53 MS/m, respectively.

**Figure 2:**
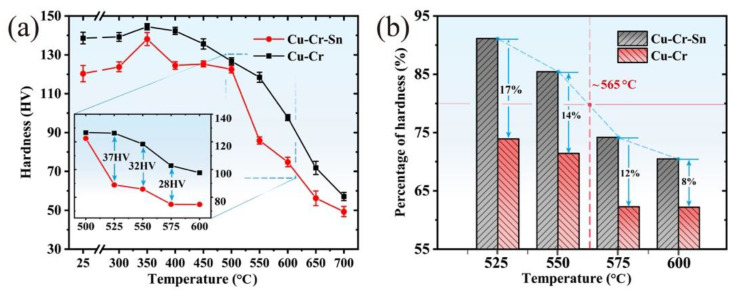
(**a**) Hardness and (**b**) percentage of hardness of Cu-Cr and Cu-Cr-Sn alloys during softening annealing.

The softening behavior curves of the peak-aged Cu-Cr and Cu-Cr-Sn alloys were obtained by holding them for 60 min at different temperatures, as shown in Figure 2a. With the increase in the softening annealing temperature, the microhardness of both alloys gradually decreases. Meanwhile, the hardness of the Cu-Cr-Sn alloy is higher than that of the Cu-Cr alloy at all temperatures. Compared to the Cu-Cr alloy, the softening rate of the Cu-Cr-Sn alloy is obviously inhibited during annealing from 500 °C to 600 °C, and the hardness increased by about 30 HV at each annealing temperature. Based on the percentage of hardness reduction in the Cu-Cr-Sn alloy during the softening process, as shown in Figure 2b, it can be seen that Sn addition significantly improves the softening resistance of the alloy. Thus, the softening temperature of the 99% cold-drawn Cu-0.2Cr-0.12Sn alloy increased to about 565 °C, according to the temperature of reduction to 80% peak-aging hardness.

### 3.2. Microstructure Evolution

**Figure 3:**
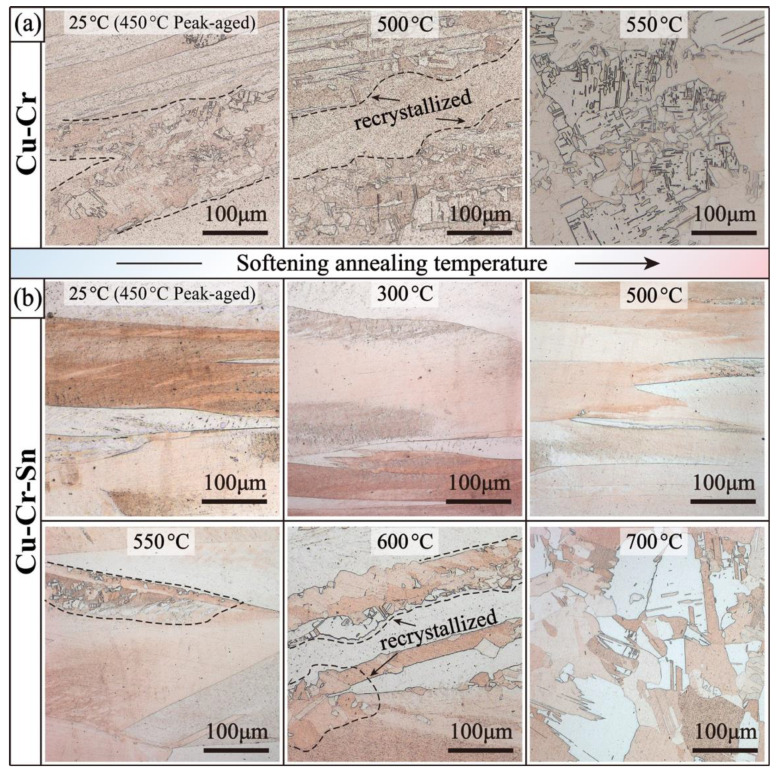
Recrystallization transition at different softening temperatures of: (**a**) Cu-Cr alloy; (**b**) Cu-Cr-Sn alloy.

Figure 3 shows the microstructure evolution of the peak-aged Cu-Cr and Cu-Cr-Sn alloys at different softening temperatures, respectively. It can be seen in Figure 3a that in the Cu-Cr alloy after annealing at 450 °C for 120 min, the recrystallization behavior occurs in many areas among the fibrous deformed grains, and the obvious equiaxed grains are found. The recovery and recrystallization processes are accelerated at higher softening temperatures. The proportion of recrystallized grain area increases significantly at 500 °C, and transformation is essentially completed during annealing at 550 °C for 60 min. In addition, the recrystallized grain size of the Cu-Cr alloy becomes much coarser and is accompanied by lots of annealing twins at 550 °C. However, the recrystallization evolution of the Cu-Cr-Sn alloy becomes much retarded, as shown in Figure 3b. After annealing from 25 °C (peak aging) to 500 °C, the Cu-Cr-Sn alloy still maintains the fibrous deformed structure without the occurrence of recrystallization. During annealing at 550 °C, only a small part of recrystallized grains is found on the grain boundaries of the deformed structure. The recrystallized grains grow further along the grain-edge boundaries during annealing at 600 °C and essentially complete the recrystallized transition at 700 °C with a lot of equiaxed grains and annealing twins formed.

**Figure 4:**
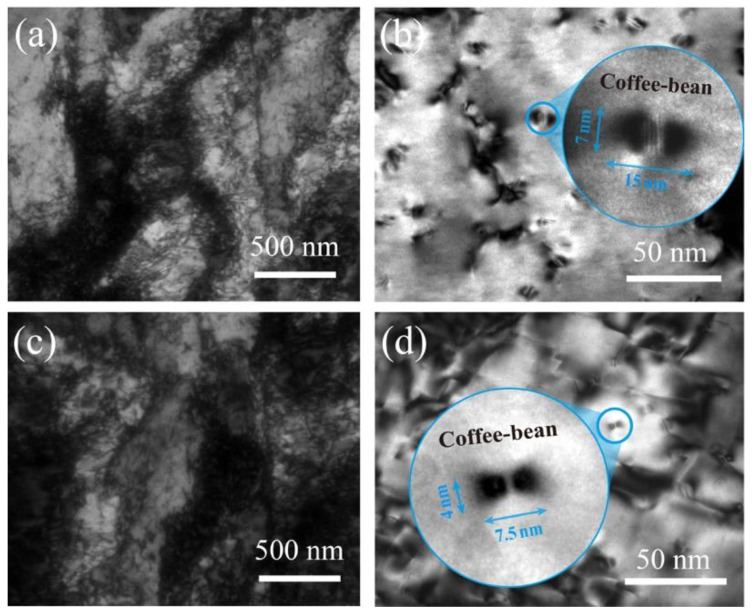
Bright-field images of (**a**,**b**) Cu-Cr and (**c**,**d**) Cu-Cr-Sn alloys after peak aging at 450 °C.

Figure 4 shows the TEM image of the peak-aged alloys during annealing at 450 °C. The high density of dislocation mutual entanglement and dislocation tangles are found in the Cu-Cr and Cu-Cr-Sn alloys after holding for 120 min and 240 min, as shown in Figure 4a,c. The nano-scale Cr-rich phases precipitated from the alloys are the main strengthening way of the Cu-Cr system alloy during aging. Figure 4b shows a large amount of Cr phases with coffee-bean and ball-like precipitates in the matrix of the Cu-Cr alloy after 120 min aging, with an average size of ~9 nm. According to the Cu-Cr-Sn alloy in Figure 4d, the Cr-rich phase still maintains a fine coffee-bean-like morphology during annealing with 240 min, and the average size is only about 6 nm.

**Figure 5:**
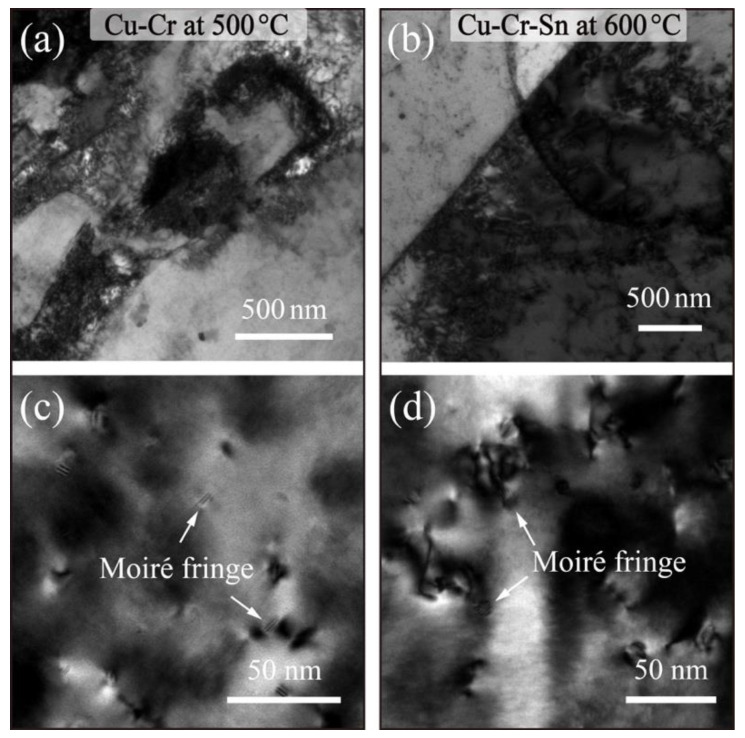
Bright-field images of softening behavior in (**a**,**c**) Cu-Cr alloy at 500 °C and (**b**,**d**) Cu-Cr-Sn alloy at 600 °C annealing for 60 min.

Dislocation multiplication after cold deformation and Cr-rich phase precipitation during aging are the critical factors of microstructural and performance regulation with the Cu-Cr alloy. However, the evolution of dislocation and substructure and the coarsening of the precipitate at high temperature lead to the rapid decreases in the strength of the Cu-Cr alloy. Figure 5 shows the BF images of the Cu-Cr and Cu-Cr-Sn alloys during annealing at their respective softening temperatures. The dislocation density in the Cu-Cr alloy is gradually annihilated after annealing at 500 °C for 60 min, as shown in Figure 5a, and rearrangement formed dislocation cell organization during the movement. When the Cu-Cr-Sn alloy was held at 600 °C for 60 min, annealing twins were observed between the dislocation structures as in Figure 5b. In addition, Figure 5c,d show the second phase particles in the alloy, and the morphology and size of the precipitated Cr phase evolved during higher temperature. The precipitated phase in the Cu-Cr alloy is mainly composed of moiré fringe and sphere-like morphologies, with an average size of ~14 nm. After annealing at 600 °C, the precipitated Cr phases with moiré fringe morphology were also formed in the Cu-Cr-Sn alloy. The average size of precipitated particles was ~15 nm, and a huge number of dislocations were entangled around the precipitated particles.

**Figure 6:**
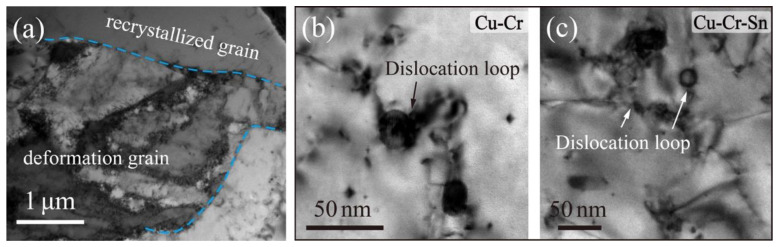
Bright-field images of (**a**,**c**) Cu-Cr-Sn and (**b**) Cu-Cr alloy after 700 °C annealing for 60 min.

Figure 6 shows the BF images of the Cu-Cr and Cu-Cr-Sn alloys after annealing at 700 °C for 60 min. It can be observed in Figure 6a that the undistorted recrystallized grains gradually replaced the deformed structure left by cold deformation and the remaining two regions in the alloy: the recrystallization zone and the deformation zone. During the softening behavior of the alloy, the fine and dispersed precipitates can inhibit dislocation and substructure transformation significantly by hindering dislocation movement. The particles of the Cr phase in the Cu-Cr alloy were obviously coarsened, with the size generally about 25 ~ 35 nm. It can also be found around the precipitated Cr phase that the dislocation bypassed and formed Orowan loops around the precipitated phase after being pinned. The precipitated Cr phase in the Cu-Cr-Sn alloy is much more stable, about 15 ~ 25 nm, and the interaction between the precipitated Cr phase and dislocations can also be observed obviously.

## 4. Discussion

As well known, Cu-Cr alloy is a typical precipitation-hardening alloy. The mechanical properties of the alloy increase rapidly during the aging process, with the transformation of the Cr phase structure. The sequence of Cr phases is solid solution → G.P zones → fcc-Cr phase → order fcc phase → bcc-Cr phase [22], and the formed stable bcc-Cr phase structure has Nishiyama–Wasserman (N-W) or Kurdjumov–Sachs (K-S) ORs with a Cu matrix [23,24]. At the same time, the fine and dispersed precipitates at high temperature can also inhibit the recovery and recrystallization transformation by the pinning effect of dislocation and substructure, which is a critical means to increase the softening resistance of the alloy [25,26]. However, the growth and coarsening of the precipitate during the over-aging stage of the Cu-Cr alloy accelerate the softening, which has a significant impact on the processing and service temperature of the alloy. Among the main micro-alloying methods, the addition of the third component improves the softening resistance of the Cu-Cr-X alloy by inhibiting the coarsening of the Cr phase [14,17], retarding structural transition [13], and forming stable compound precipitates with Cr [27,28]. In this study, the addition of Sn to the Cu-Cr alloy can improve the mechanical properties of the Cu-Cr alloy during aging and shows outstanding thermal stability at high temperature.

The peak hardness of the Cu-Cr-Sn alloy increased by about 19 HV compared with the Cu-Cr alloy, which shows good mechanical properties after prolonged annealing, as shown in Figure 1. However, the hardness of the Cu-Cr alloy begins to decrease rapidly after annealing at 450 °C for 120 min, because of a large region of recrystallization formed in the alloy, while the Cu-Cr-Sn alloy maintains the fibrous deformed structure left by cold drawing completely (Figure 3). The addition of the Sn element can effectively promote the precipitation of the Cr phase during the aging process [20] and precipitate finer Cr phase particles in the matrix dispersedly. Both the size and structure of the Cr-rich phase are still stable in the Cu-Cr-Sn alloy after holding for 240 min (with average ~ 6 nm), which is smaller than the Cu-Cr alloy aged for 120 min (Figure 4). The addition of Sn can inhibit the growth and coarsening of the precipitated Cr phase, which improves the thermal stability of the Cu-Cr alloy during aging.

**Figure 7:**
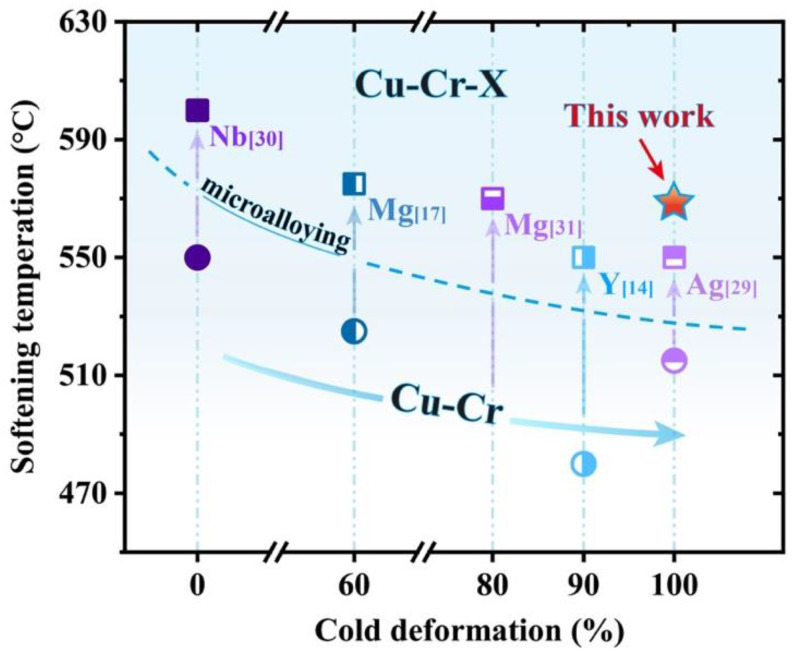
Relationship between softening temperature and cold deformation of Cu-Cr-(X) alloys.

In a further study of the softening behavior of the two alloys, it can also be found that the Cu-Cr-Sn alloy retained the positive mechanical properties from 500 °C to 600 °C. After annealing at various temperatures for 60 min, the recrystallization of the Cu-Cr-Sn alloy occurred until 550 °C, while the Cu-Cr alloy completely transformed into an equiaxed grain structure at this temperature (Figure 3). The precipitated phase of the Cu-Cr-Sn alloy still maintained a fine moiré fringe and sphere-like morphology after annealing at 600 °C for 60 min, with an average of ~12 nm (Figure 5). The fine and dispersive precipitated Cr phase can greatly delay the transformation of the substructure, which also makes the Cu-Cr-Sn alloy maintain a large area of deformed microstructure at 600 °C (Figure 3). The precipitated phase of the Cu-Cr-Sn alloy was coarsened to a certain extent at 700 °C, but the average size is smaller than that of the Cu-Cr alloy, and some deformation structure, dislocation cell structure, is still retained in the microstructure (Figure 7). The element of Sn inhibits the coarsening of the precipitated phase and delays the recrystallization transition of the alloy, which contributes to the improvement of softening resistance in this work.

The softening temperature of the Cu-Cr-Sn alloy is about 565 °C, which is much higher than that of the Cu-Cr alloy with 99% cold work. With the increase in cold deformation degree, the activation energy of recrystallization is decreased as well as the work hardening at high temperatures. As shown in Figure 7, the major microalloying element additions can significantly suppress the softening behavior of the Cu-Cr alloy [29,30]. The softening temperature of the 90% cold-rolled Cu-Cr-Y alloy is about 550 °C and higher than the Cu-Cr alloy [14]. Additionally, the softening temperature of the 99% cold-drawn Cu-Cr-Ag alloy also increased from 515 °C to 550 °C by Ag addition [31].

## 5. Conclusions

In the present study, the aging precipitation behavior and recrystallization evolution process of Cu-0.2Cr and Cu-0.2Cr-0.12Sn alloys were investigated in detail. The main results show that:

1. The peak hardness of the Cu-Cr and Cu-Cr-Sn alloys reached 120 HV and 139 HV after aging at 450 °C for 120 min and 240 min, respectively. With the extension of aging time, the addition of Sn can also obviously enhance the thermal stability of the alloy.

2. During the process of annealing at high temperature, the Sn element inhibits the growth and coarsening of the Cr-rich phase in the alloy, which delays the recovery and recrystallization transition and significantly improves the thermal stability of the Cu-Cr-Sn alloy. Moreover, the softening temperature of the 99% deformation Cu-0.2Cr-0.12Sn alloy can be confirmed as 565 °C.

## Figures and Tables

**Table 1:** The chemical compositions of Cu-Cr and Cu-Cr-Sn alloys (wt.%).

Elements		Cr	Sn	Cu
Measured composition	Cu-Cr	0.20	—	Bal.
	Cu-Cr-Sn	0.20	0.12	Bal.

## Data Availability

Not applicable.
